# Ecotoxicological Effects of Potassium Dichromate on the Tadpole Shrimp *Triops longicaudatus*

**DOI:** 10.3390/ani14030358

**Published:** 2024-01-23

**Authors:** André Carido Pereira, Aurélia Saraiva, Luís Oliva-Teles, Laura Guimarães, António Paulo Carvalho

**Affiliations:** 1 CIIMAR—Interdisciplinary Centre of Marine and Environmental Research, University of Porto, 4450-208 Matosinhos, Portugal; andre.cpereira92@gmail.com (A.C.P.); amsaraiv@fc.up.pt (A.S.); loteles@fc.up.pt (L.O.-T.); 2 Biology Department, FCUP—Faculty of Sciences, University of Porto, 4169-007 Porto, Portugal; 3 ICBAS—School of Medicine and Biomedical Sciences, University of Porto, 4050-313 Porto, Portugal

**Keywords:** triops, hexavalent chromium, locomotor behavior, cholinesterase activity

## Abstract

**Simple Summary:**

Over the years, research from other areas has suggested that tadpole shrimps could be a useful model to detect and evaluate the effects of aquatic contamination and environmental health. Its size, active swimming, short generation time, and easy lab maintenance make it an attractive alternative to the use of higher sentient animals. This investigation has thus aimed at evaluating the sensitivity of *Triops longicaudatus* to potassium dichromate, a common contaminant in aquatic systems, also used as reference compound in ecotoxicology. Lethal and sublethal exposure assays were carried out and biochemical to apical endpoints with an impact at the population level were measured. We found negative impacts in the growth rate, delayed reproductive maturity, and alteration in the locomotor behavior upon exposure to potassium dichromate. The sensitivity of the species was higher than that of various freshwater animals commonly used in toxicity testing. Behavior appeared as the most sensitive response to chromium exposure, discriminating well the test concentrations from the control group and from each other.

**Abstract:**

The tadpole shrimp *Triops longicaudatus* is a freshwater crustacean with fast embryonic and larval development, short life cycle, and high fecundity. They are very active swimmers of a reasonable size, easy to spot and record. Such characteristics make it a promising candidate as an experimental model in ecotoxicology to evaluate the effects of aquatic pollutants, particularly using its locomotor behavior as an endpoint. To evaluate the sensitivity of *T. longicaudatus* and develop endpoints of interest, we conducted exposure experiments with lethal and sub-lethal concentrations of potassium dichromate, a compound known for its ecotoxicological importance and as a hexavalent chromium source. The endpoints evaluated were mortality, growth, sexual maturation, reproductive output, cholinesterase activity and locomotor/swimming behavior. The 96 h median lethal concentration was found to be 65 µg/L. Furthermore, exposure to potassium dichromate at higher concentrations had a significant negative impact on the growth rate of *T. longicaudatus* in terms of both body mass and length. The time for maturation was also delayed at higher concentrations. In addition, locomotor behavior allowed for the discrimination of all tested chromium concentrations and the control group and from each other, proving to be the most sensitive endpoint. Overall, the data support the potential of *T. longicaudatus* as a model for ecotoxicity testing, using apical endpoints with impact at the population level; in particular, results suggest that behavior assessments in this species might be useful for detecting hazardous compounds in environmental monitoring of freshwater ecosystems.

## 1. Introduction

The genus *Triops* comprises a number of species of widely distributed freshwater branchiopod crustaceans, commonly referred to as triops or tadpole shrimps, known to inhabit ephemeral shallow water bodies. Triops exhibit several reproductive modes [1], and populations of the same species are often found reproducing differently, either sexually or by parthenogenesis. These crustaceans show high fecundity, producing thousands of eggs throughout their lifespan, often in clutches exceeding 150 eggs [2,3]. Eggs are carried in pouches and oviposition is preceded by burrowing shallow chambers in the substrate, where the successive egg clutches are laid [3,4]. The survival of triops species in temporary water bodies comes from their ability to produce desiccation-resistant eggs during the dry periods, known as cysts or dormant/resting eggs; these remain viable in the sediment, with suspended development for long periods (up to several years), until their rehydration at the next flooding of the habitat, when they hatch [5]. Triop cysts can be easily obtained from laboratory cultures of these animals, by collecting eggs from the substrate three days after oviposition (time required for complete embryonic development) followed by their dehydration/drying [6]; for some species, such as *Triops longicaudatus*, dehydrated cysts are also commercially available, similarly to brine shrimp *Artemia* cysts. Cysts hatch after one to three days of rehydration; a rapid larval development then takes place, comprising five planktonic and filter-feeding larval stages, which results in a benthic juvenile with predatory behavior, similar to the adult [7]. Sexual maturation is also reached rapidly, as early as 7 days after hydration at 30 °C in *T. longicaudatus* [6].

From a technical and scientific perspective, *T. longicaudatus* has specific characteristics that make it an excellent potential model for ecotoxicological studies, particularly using locomotor behavior as an endpoint. In fact, a short life cycle, fast embryonic and larval development, high fecundity and intense locomotor behavior (i.e., swimming, digging) are considered advantageous biological features for ecotoxicity testing. Additionally, the ability of this species for parthenogenetic reproduction and production of cysts facilitates its large-scale breeding within a short time frame, ruling out the need for a permanent animal stock. However, there are few toxicological studies with triops. Most of these studies are old and related to the emergence of triops as a pest in rice fields, investigating chemicals that could prevent their proliferation and thus, the damage they cause to rice seeds and plants [8,9,10], or to the role of triops as a biological agent in controlling mosquito populations in ponds, seeking effective concentrations of mosquito larvicidal chemicals without adverse effects on triops to make the use of both compatible [11,12]. Actually, the potential of *T. longicaudatus* as a model for ecotoxicological assays has only recently been highlighted [13,14].

To further investigate the sensitivity of the species, and develop endpoints of interest, this study evaluated the effects of potassium dichromate on *T. longicaudatus*. Potassium dichromate is a reference toxicant in ecotoxicological studies and standardized guidelines, used as a source of hexavalent chromium [Cr (VI)] [15,16]. Chromium is one of the most frequent and pervasive contaminants in the environment and does not exist in its pure metallic form in nature. It is present in the environment in its oxidation states: bivalent [Cr(II)], trivalent [Cr(III)], and hexavalent [Cr(VI)]. Trivalent and hexavalent chromium are the most common and stable forms [17,18]. Chromium has several applications in chemical, metallurgical and refractory industries, particularly in tanning, corrosion inhibition, electroplating, solutions for glass cleaning, preservation of wood, production of safety matches, pigments and metal finishing [17]. Anthropogenic sources, in addition to natural ones, release tri- and hexavalent chromium into the environment. Exposure to chromium can be a significant health hazard, depending on the oxidation state of the metal. The toxicity of the trivalent form is considered low and naturally present in biological materials. The hexavalent form is highly toxic to aquatic animals and humans, as well as clastogenic and carcinogenic [18,19]. Hexavalent chromium can also induce histological changes in crustaceans, such as *Palaemonetes pugio* [20], behavioral alterations in invertebrates [21] and fish [22,23], decreased appetite [24], as well as developmental effects and metabolic alterations [25,26], genotoxicity and DNA damage [25,27]. Hexavalent chromium is also neurotoxic to humans and several aquatic species, including zebrafish *Danio rerio* [23,28,29,30].

This work has thus investigated the effects of potassium dichromate on *T. longicaudatus* growth, reproduction, sexual maturation, and locomotor behavior, as well as on cholinesterase activity (ChE) to assess the feasibility of biochemical evaluations. Behavioral endpoints have emerged over the last decades as a key tool for assessing effects of toxicants, without the requirement of invasive methodologies. The significance of this approach stems from the fact that an organism’s behavior is influenced by its immediate surroundings and its own health status, including the available energy, which are ultimately influenced by exposure to toxicants. An array of possible behaviors can be assessed in animals in general, including balance, respiration, locomotion, mating, fear reactions, and righting ability. Among these, locomotion comes out as particularly sensitive to chemical exposure, compared to apical endpoints [31,32]. Though it is assessed at the individual level, locomotion is considered to have a direct reflex on populations due to its involvement on feeding, prey escaping and mating, which renders it valuable as an early warning indicator of toxicant exposure [31]. On the other hand, the activity of the acetylcholinesterase enzyme is a common biomarker of neurotoxicity, linked to neuromuscular stimulation involved in locomotion. For this reason, it is widely employed to assess exposure to and effects of chemical contamination in laboratory investigations and biomonitoring programmes [33,34]. This enzyme allows for normal nerve function through its role on the degradation of the neurotransmitter acetylcholine in the cholinergic synapses [35]. Importantly, though the mechanisms through which this occurs are not clear yet, hexavalent chromium has been shown to decrease acetylcholinesterase activity, as reviewed by Wise et al. [30]. According to the authors, the mechanistic data indicates that Cr(VI) would enter cells via phosphate or sulphate channels [30]. Rapid reduction into Cr(III) with production of reactive oxygen species would then occur in the cytoplasm, possibly triggering a series of reactions including: decreased antioxidant defenses (i.e., glutathione levels), increased oxidative damage (lipid peroxidation), increased expression of stress response genes and decreased levels of acetylcholinesterase activity.

## 2. Materials and Methods

### 2.1. Chemicals and Test Solutions

Potassium dichromate (K_2_Cr_2_O_7_; CAS No.: 7778-60-9, ≥99.98%) was acquired from Sigma-Aldrich (St. Louis, MO, USA). A stock solution was prepared by dissolving potassium dichromate in deionized water. The test solutions were obtained by diluting the former in dechlorinated tap water.

### 2.2. Test Organisms

Assays were conducted using laboratory reared parthenogenetic *T. longicaudatus* maintained at our facilities (CIIMAR, University of Porto, Porto, Portugal) for at least three generations. The original cysts were acquired from “Triops king-Germany”. Fine sand containing *T. longicaudatus* eggs was placed in polyethylene containers of 10 cm diameter and 4.5 cm height, filled with 250 mL of dechlorinated tap water. To stimulate hatching, the containers were subjected to constant light conditions at a temperature of 25 ± 1 °C. After hatching, the photoperiod was modified to 14 h light and 10 h dark, and animals were raised to the juvenile stage according to a protocol we have been implementing in our laboratory. Briefly, nauplii in each container were fed a daily dose of 20 mg of ground *Spirulina*-based granulate until the third day post-hatching, when half of the medium was replaced. On the fourth day, animals were counted and started to be fed with a fish granulate, at an initial rate of 1 mg per individual; on subsequent days the feeding rate was increased by 1 mg per individual per day, and the medium was renewed daily. On the seventh day, groups of 14 animals (juveniles) were taken from the initial containers and transferred to borosilicate glass vessels with (15 × 15 × 4.5 cm), filled with 800 mL of dechlorinated tap water. From the seventh to the tenth day, temperature was lowered to 23 ± 1 °C, since at this temperature we have observed a reduction in the cannibalistic behavior of these animals, probably due to a metabolic slowdown.

### 2.3. Exposure Assays

A preliminary assay was initially conducted to obtain some information on the lethal median concentration (LC_50_) and plan the subsequent assays. For this preliminary assay, test concentrations were decided based on the available data for other freshwater species and were 0, 30, 60, 120 and 240 µg/L of potassium dichromate. For the exposure, 10 day-old triops were randomly distributed in glass test vessels (19 × 19 × 5 cm), each containing 8 individuals and filled with 1.5 L of dechlorinated tap water (control groups) or the potassium dichromate test solutions. Four replicates were prepared per test condition, in a total of 32 individuals per treatment. The duration of the assay was 96 h. The test vessels were kept in a culture chamber (Velp Scientifica FOC215I, Usmate Velate, Italy) throughout the exposure, at 23 ± 1 °C and 14 h light/10 h dark. Relevant water parameters were monitored daily: pH (with a pH meter, PH 20, VWR, Leuven, Belgium), ammonia (by the Nessler method, with a Multiparameter Photometer 83399, HANNA Instruments, Woonsocket, RI, USA) and dissolved oxygen (with a dissolved oxygen meter, DO 40, VWR, Leuven, Belgium). Mortality was recorded daily, followed by removing dead animals and exuviae, medium renewal, and feeding.

Based on the results of the preliminary acute assay, the following concentrations were tested in a 144 h assay for evaluation of sublethal effects: 0, 10, 20, 40 and 80 µg/L of potassium dichromate. The exposure conditions were the same as in the acute toxicity test. Mortality, amount of ingested feed, number of exuviae and matured animals (appearance of ovisacs) were recorded daily. At the beginning and end of the 144 h exposure period, in each replicate of all treatments, triops were photographed for body length measurement (using the ImageJ2 FIJI software, https://fiji.sc/, accessed on 1 December 2023) and the total biomass (fresh weight) was recorded. A number of parameters were calculated using the following formulas:

Feed intake (mg/g ABW/day) = mg of ingested feed/g of average body weight/number of days, where average body weight (ABW) = (initial body weight + final body weight)/2

Body Length Growth (%) = [(final body length − initial body length)/initial body length] × 100

Specific Growth Rate (SGR) = [(ln(final body weight) − ln(initial body weight))/number of days] × 100

At the end of the exposure all animals underwent a video-tracking evaluation of the locomotor behavior. Following the behavior evaluation, part of the animals was used to assess the post-exposure reproductive output and another part was sampled for the biochemical assessment of cholinesterase activity.

### 2.4. Locomotor Behavior Analysis

The video-recording setup used was similar to the one previously described by Amorim et al. [36]. Immediately after the end of the sub-lethal exposure, triops were filmed for a total of 25 min. The first 5 min allows the tadpole shrimps to acclimate to the arenas, and the remaining 20 min of recording are used for behavioral analysis.

Each individual was placed in an opaque plastic container (arena) (circular, food grade, polypropylene, diameter 10 cm, height 4.5 cm) with 200 mL of dechlorinated tap water. Recordings were performed using four Flow Electronics 540L IR (model: CACO0008) cameras connected to Camtronics DVR 38 AHD PLUS capture device (Leganes, Spain). All animals from each experimental condition were randomly distributed in the arenas (nine arenas per camera for a total 36 arenas per recording). The recording chamber was isolated with expanded cork (20 mm thick), to minimize external interference such as vibrations, external lights and noise.

Recorded videos were then processed using an adapted open-source algorithm described by Guimarães et al. [14], for MatLab software (https://www.mathworks.com/products/matlab.html, accessed on 1 December 2023), which is available upon request. The adapted algorithm is based on multiwellTracker [37] and allows for the extraction of six behavior parameters: mean velocity (mm/s), mean angular velocity (degrees/s), instantaneous velocity (mm/s), mean meander (degrees/mm, degree of curvature by unity of displacement), instantaneous acceleration (mm/s^2^), and distance to the center of the arena (mm).

### 2.5. Post-Exposure Reproductive Assessment

Following the video recording, a seven-day reproduction assay was performed. Three mature triops from each treatment were randomly selected and individually transferred to glass containers (19 × 19 × 5 cm) filled with 1.5 L of dechlorinated tap water. Each container featured a single layer of glass beads (8 mm Ø) on the bottom to allow eggs to fall through the slits, avoiding potential egg predation. A small Petri dish (55 mm Ø) was placed between the glass beads, where feed was deposited. Triops were fed daily following the medium renewal. Eggs were sieved, counted, and stored daily.

### 2.6. Cholinesterase (ChE) Activity Assessment

A total of 10 individuals from each experimental condition were randomly selected and stored at −80 °C for subsequent determination of ChE activity. Whole animal bodies were homogenized in potassium phosphate buffer (0.1 M, pH 7.2), and ChE activity was assayed in the supernatant that was obtained following centrifugation (4 °C, 6000× *g*, 5 min). Total ChE activity was measured using the Ellman method [38] as described previously [39]. For this, the homogenate was incubated with a reaction solution containing 10 mM 5,50-dithiobis-2-nitrobenzoate and 0.075 M acetylthiocholine in 0.1 M phosphate buffer. The increase in absorbance caused by the hydrolyzation of acetylthiocholine was measured at 414 nm. Triplicate measurements were performed per sample, and the results were reported as nanomoles of substrate hydrolyzed per minute per mg of protein. The Bradford method [40] was employed to measure the protein content in the samples, with bovine γ-globulin as standard.

### 2.7. Statistical Analysis

Following the Shapiro–Wilk normality test and the Levene homoscedasticity test, data was analyzed employing one-way analysis of variance (ANOVA) for normal data sets, and non-parametric Kruskal–Wallis for non-normal data sets. Tukey multiple range test was used to distinguish between means when significant differences were found in the ANOVA (*p* < 0.05), whereas a Dunn’s pairwise test with Bonferroni correction was performed when significant differences were detected by the Kruskal–Wallis test (*p* < 0.05). Probit analysis was used to determine the median lethal concentration (LC_50_) and the median time to maturation (TM_50_). The analyses were performed in IBM SPSS v24. Behavioral data was analyzed using a Cluster Analysis with an artificial neuronal network (ANN) algorithm as described in a previous study [32], to define six main behavioral types. One-way ANOVA followed by the Tuckey HSD Test was then used to investigate differences among the behavioral types. Differences across experimental conditions were investigated with a chi-square test with behavior types and chromium treatments as independent factors. Specific differences among toxicant treatments were identified through a residual analysis. The statistical significance of each residual was determined by comparing the respective partial chi-square value with the critical distribution value determined with the Bonferroni correction for the total number of cells. The analysis was performed with Statistica 14.0.0.15v.

## 3. Results and Discussion

Potassium dichromate (K_2_Cr_2_O_7_) is considered very harmful to freshwater species such as fish and invertebrates. It may cause considerable damage to aquatic species at molecular, histophysiological, and population levels [22,27]. In the present study, *T. longicaudatus* juveniles were exposed to several concentrations of K_2_Cr_2_O_7_. During the toxicity tests, pH ranged from 6.8 to 7.2, the highest recorded level of ammonia was 0.06 mg/L and the lowest recorded dissolved oxygen was 6.3 mg/L. The maximum mortality recorded in the control was 10%. The estimated LC_50_ values ranged from 181 µg/L at 24 h to 65 µg/L at 96 h of exposure (Table 1).

Previous studies found higher LC_50_ values of potassium dichromate in other test species, including rotifer, amphipod and cladoceran species (Table 2), which indicates greater sensitivity of *T. longicaudatus* to this reference toxicant. The effect concentrations found in this work were in the range or lower than pure hexavalent chromium concentrations detected in environmental water samples, which can be as high as 200 µg/L of pure Cr(VI) [41,42,43], often exceeding the provisional guideline value of total chromium (50 µg/L) [44].

**Figure 1:**
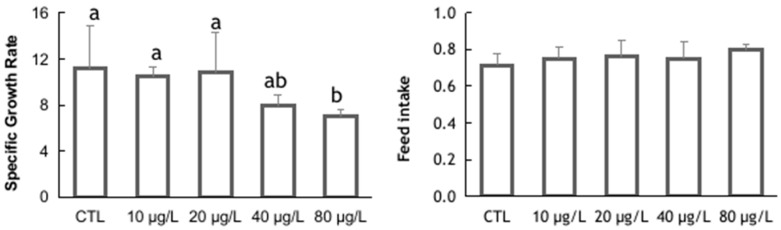
Specific growth rate (%/day) and feed intake (mg/g ABW/day) determined in *Triops longicaudatus* exposed to potassium dichromate for 144 h. Values represent the group means and the respective standard deviations. Different letters indicated statistically significant differences (Kruskal–Wallis test followed by Dunn’s pairwise test with Bonferroni correction, *p* < 0.05).

In the sublethal toxicity assay, the mortality in the control group was 12.5% and in the exposed groups was consistent with the estimated LC_50_. A significant decrease in the SGR was recorded in animals exposed to the highest toxicant concentration (Kruskal–Wallis: H = 10.47; *p* = 0.033) (Figure 1). Since feed intake appeared to be unaffected by exposure to potassium dichromate (ANOVA: F(4,15) = 0.447; *p* = 0.773) (Figure 1), it can be ruled out that the lower growth observed in animals exposed to higher toxicant concentrations is due to a decrease in feed consumption.

**Figure 2:**
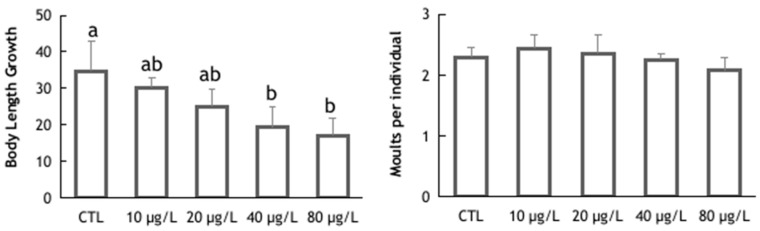
Body Length Growth (%) and total number of molts in *Triops longicaudatus* exposed to potassium dichromate for 144 h. Values are presented as group means and respective standard deviations. Different letters indicated statistically significant differences (ANOVA followed by Tukey’s test, *p* < 0.05).

**Figure 3:**
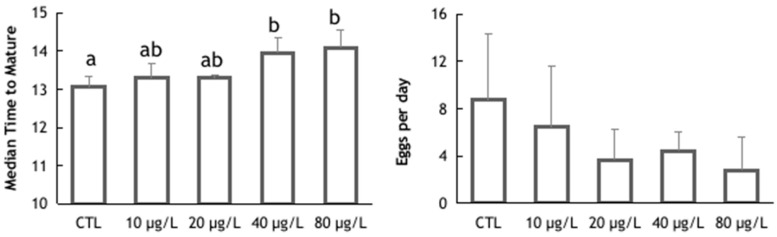
Time to maturation (days) and mean number of eggs laid daily in *Triops longicaudatus* exposed to potassium dichromate for 144 h. Values are presented as group means and their standard deviation. Different letters indicated statistically significant differences (ANOVA followed by Tukey’s test, *p* < 0.05; Kruskal–Wallis test, *p* < 0.05).

In addition to a decrease in body weight gain, potassium dichromate also affected triops body length growth. Significant differences were detected between the control group and the two highest potassium dichromate concentrations (ANOVA: F(4,15) = 5.401; *p* = 0.007) (Figure 2). Despite the detected differences in length, no differences were observed in the number of molts per individual between exposure concentrations (ANOVA: F(4,15) = 1.186; *p* = 0.357) (Figure 2). Even though no statistically significant variations were found, the number of molts per individual could be an important endpoint for future assays, given the fact that the process of molting is essential for growth and development in this species, and as such needs to be considered. Control triops molted every 2.6 days. This is similar to what was observed in an earlier study [49], where *T. longicaudatus* molted every 2.5 to 2.8 days. Triops exposed to higher concentrations of potassium dichromate (40 and 80 µg/L) showed an increase in the time to maturation compared to the control (way ANOVA: F(4,15) = 5.138; *p* = 0.008) (Figure 3). All animals reached maturity (appearance of ovisacs) within 14.5 days in the control group (TM_50_:13.1 days; 95% CI: 12.7–13.4), while there were still immature animals up to 16 days of age in the groups exposed to the two highest toxicant concentrations. Although the time for maturation was affected by the exposure to potassium dichromate, no differences among the experimental treatments were found for the number of eggs laid per individual (Kruskal–Wallis: H = 3.87; *p* = 0.424), despite the general trend for a decrease in the average number of eggs with increasing toxicant concentration (Figure 3). The delayed reproductive maturity may be related to the lower growth rate of triops exposed to the highest concentrations of potassium dichromate. Effects of potassium dichromate in the reproductive output were reported, however, for other species; for example, lower reproductive output in *D. magna* [47] and in *D. schodleri* [50].

**Figure 4:**
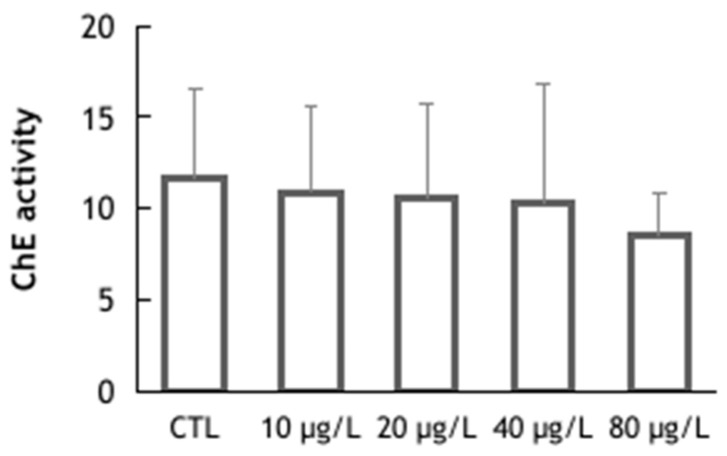
Cholinesterase activity (nmol/min/mg protein) determined in *Triops longicaudatus* exposed to potassium dichromate for 144 h. Values represent the group means and respective standard deviations in nmol/min/mg protein (Kruskal–Wallis, test *p* < 0.05).

As to ChE activity, the baseline level in the control group was 11.6 ± 4.9 nmol/min/mg protein. This is comparable to the levels observed in other branchiopods, such as *D. magna* (9.3 ± 1.02 nmol/min/mg protein) [51], and in isopods, such as *Porcellio dilatatus*, (12.1 ± 1.92 nmol/min/mg protein) [52]. The results also revealed no significant differences in ChE activity among the experimental treatments (Kruskal–Wallis: H = 2.12; *p* = 0.714) (Figure 4). Similar results were reported in the work presented in [53], which evaluated the effects of sodium dichromate in *D. magna*, though their exposure time was much shorter (48 h vs. 144 h in the present work) and the test concentrations were higher (12.5 to 100 mg/L).

**Figure 5:**
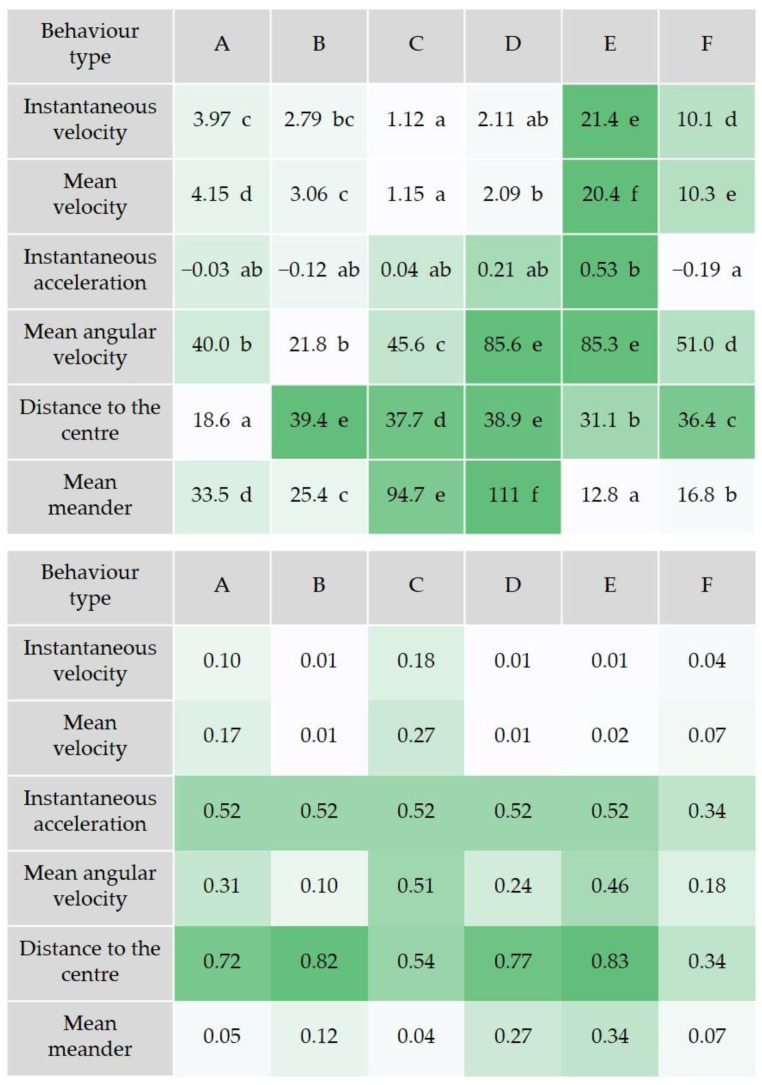
Characteristics of the behavior types defined by the Cluster Analysis based on the Artificial Neural Network. (**Top**) average values of the most relevant movement parameters in each behavior type (different letters indicate significant differences at *p* < 0.05 as identified through the post hoc analysis). (**Bottom**) contribution (weight) of each movement parameter to the different behavior types. The darker the green shade, the stronger the contribution. The results are grouped according to similarities in behavioral types across toxicants as found through cluster analysis.

**Figure 6:**
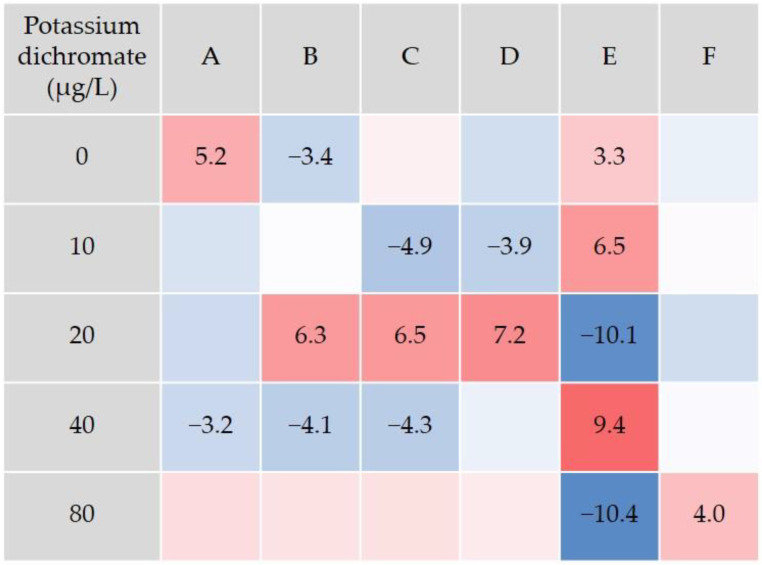
Results of the residual analysis performed to identify significant differences among the experimental treatments. Only the significant residuals are shown in the table; statistical significance of each residual was determined by comparing the respective partial chi-square value against the critical distribution value estimated with the Bonferroni correction for a significance level of 0.05. Blue and red shades indicate negative or positive residuals, respectively; the darker the shade, the stronger the deviation.

The cluster analysis based on the ANN defined six different behavior types (A to F, Figure 5). This analysis was performed with all the movement data obtained, irrespective of the experimental treatment. The defined behavioral types reflected a gradient of variation to which the movement variables contributed differently, resulting in distinctive profiles. The average values and contribution to each behavior type are shown in Figure 5. Instantaneous acceleration and distance to the center of the swimming arena were the parameters with highest weight on the definition of the behavioral types (Figure 5, bottom). Based on these, each behavioral type showed a characteristic locomotion. When exhibiting behavior type E, for instance, the animals tended mainly to change a lot their swimming velocity, rotate fast and swim fast at mid to high distance from the center of the arena, as indicated by the variables instantaneous acceleration, mean angular velocity and distance to the center, respectively. They also tended to show high velocity and straight swimming route (few curving), as indicated by the high instantaneous velocity and the low mean meander. Animals exhibiting behavior type D, tended to change less their swimming velocity, rotate fast, swim in the periphery and curve a lot (i.e., winding swimming route, Figure 5). Animals with prominent behavior type A, tended overall to show a steady movement with little change in velocity and swimming near the center of the arena (Figure 5). Further analysis of differences among treatments elicited by exposure to chromium dichromate were identified through the chi-square analysis. Globally, the control group tended to show significantly higher frequency of behavior types A and E, and lower frequency of behavior type B (Figure 6). Behavior types B to F further distinguished the chromium treatments from the control group and from each other. In contrast with the control group, the lowest chromium concentration (10 µg/L) tended to show significantly decreased frequencies of behavior types C and D, and increased frequency of behavior type E (Figure 6). The strongest changes in the behavior types were found for the concentrations ≥ 20 µg/L. They were observed, in particular, in behavior type E, which was found to decline markedly below average in the 20 and 80 µg/L groups, and increased in frequency in triops exposed to 40 µg/L. Behavior type F was at average level in all groups, except for the highest test concentration (80 µg/L). At this exposure level, triops tended to show an increase in F frequency that contributes to distinguish this treatment from the remaining. Studies assessing the behavior of aquatic invertebrates as a toxicity endpoint for exposure to potassium dichromate are scarce. Nevertheless, Gutierrez et al. [54] detected changes in diel vertical migration (DVM) in five freshwater zooplankton crustaceans (*Argyrodiaptomus falcifer*, *Notodiaptomus conifer*, *Pseudosida variabilis*, *Ceriodaphnia dubia* and *Daphnia magna*), upon exposure to chromium in the form of potassium dichromate. Each species was exposed for 24 h to a low and a high chromium concentration while DVM was assessed. Depending on the species, the low concentration ranged from 3.5 to 15 µg/L, while the high concentration ranged from 14 to 60 µg/L of chromium. The authors hypothesized that reduced swimming activity and disorientation were the underlying causes for DVM changes observed. Exposure to 8.5 µg/L chromium for 24 h also affected the ability to escape predation in the copepod *N. conifer* [55]. The authors found that chromium elicited higher “capturability” of the exposed animals. Overall, the present results raise special concern, since reduced growth was found in the exposed animals, together with delayed reproductive maturation and alterations of the swimming behavior. The behavior alterations observed may affect the population by interfering with prey-predator interactions, for example by making animals easier to spot. The data also supports the species potential, ecological parameters measured and the sensitivity of the behavioral approach to discriminate the negatively impacted animals exposed to chromium from the control animals.

## 4. Conclusions

The present study revealed that potassium dichromate can cause toxic effects on *T. longicaudatus*. Exposure to this source of hexavalent chromium reduced survival and growth, increased the time to attain reproductive maturation, and affected the locomotor behavior of the animals. Further studies should be carried out to assess other important endpoints such as genotoxicity, histological effects and biochemical markers involved in detoxification and antioxidant defenses (e.g., glutathione s-transferase activity, catalase activity, lipid peroxidation). The results suggest that behavior (the most sensitive endpoint) assessments in *T. longicaudatus* might be useful for environmental monitoring of ecosystem quality due to their sensitivity to reference compounds. Furthermore, some of the effects of Cr(VI) described herein, in the form of potassium dichromate, occurred at concentrations found in environmental samples, raising serious concern about possible harm to aquatic ecosystems.

## Figures and Tables

**Table 1:** Lethal concentration values (LC_50_ and LC_10_) of potassium dichromate and respective confidence intervals (CI) for *Triops longicaudatus* exposed up to 96 h.

Hours	LC_50_ (µg/L)	CI 95% (µg/L)	LC_10_ (µg/L)	CI 95% (µg/L)
24	181.4	154.2–222.0	95.0	65.7–116.1
48	81.4	70.3–94.3	46.9	34.9–56.2
96	65.3	57.6–74.9	43.4	33.8–50.2

**Table 2:** Median lethal concentration (LC_50_) or effective concentration (immobilisation, EC_50_) values and respective confidence intervals (CI) of potassium dichromate reported in the literature for other invertebrate species.

Species	Hours	LC_50_/EC_50_ (µg/L)	CI 95% (µg/L)
*Philodina roseola*	48	47,100 *	29,520–64,670 [45]
*Daphnia pulex*	48	509.2	340–734 [46]
	96	198.1	85–283 [46]
*Daphnia carinata*	48	396.1	255–594 [46]
	96	198.1	85–283 [46]
Daphnia magna **	48	3451.3	2263–6224 [46]
	96	1725.6	1075–2546 [46]
	48	494 *	457–532 [47]
*Ceriodaphnia quadrangular*	48	537.5	368–764 [46]
	96	254.6	170–368 [46]
*Bosmina longirostris*	48	480.9	311–736 [46]
	96	169.7	85–283 [46]
*Simocephalus vetulus*	48	763.8	509–1216 [46]
	96	396.1	226–622 [46]
*Hyalella curvispina*	96	1555.9	1330–1839 [48]
	96	565.8	424–792 [48]

* immobilization, EC_50_. ** exposure of 2-day-old cladocerans in [46] and neonates < 24-h old in [47].

## Data Availability

Data can be shared upon request.

## References

[1] Sassaman C. (1991). Sex ratio variation in female-biased populations of Notostracans. Hydrobiologia.

[2] Takahashi F. (1977). Pioneer life of the tadpole shrimps, *Triops* spp. (Notostraca:Triopsidae). Appl. Entomol. Zool..

[3] Meintjes S. (1996). Observations on some reproductive traits of *Triops granarius* (Lucas) (Crustacea: Notostraca). Hydrobiologia.

[4] Longhurst A.R. (1955). A review of the Notostraca. Bull. Br. Mus. Nat. Hist. Zool..

[5] Su T., Mulla M.S. (2002). Factors affecting egg hatch of the tadpole shrimp, *Triops newberryi*, a potential biological control agent of immature mosquitoes. Biol. Control.

[6] Fry-O’Brien L., Mulla M. (1996). Optimal conditions for rearing the tadpole shrimp, *Triops longicaudatus* (Notostraca:Triopsidae), a biological control agent against mosquitoes. J. Am. Mosq. Control Assoc..

[7] Davis J., Madison D. (2000). The ontogeny of light-dark response in *Triops Longicaudatus* as a response to changing selective pressures. Crustaceana.

[8] Grigarick A.A., Lange W.H., Finfrock D.C. (1961). Control of the tadpole shrimp, *Triops longicaudatus*, in California rice fields. J. Econ. Entomol..

[9] Walton W.E., Darwazeh H.A., Mulla M.S., Schreiber E.T. (1990). Impact of selected synthetic pyrethroids and organophosphorous pesticides on the tadpole shrimp, *Triops longicaudatus* (Le Conte) (Notostraca:Triopsidae). Bull. Environ. Contam. Toxicol..

[10] Tsukimura B., Nelson W.K., Linder C.J. (2006). Inhibition of ovarian development by methyl farnesoate in the tadpole shrimp, *Triops longicaudatus*. Comp. Biochem. Physiol.-A Mol. Integr. Physiol..

[11] Su T., Mulla M.S. (2005). Toxicity and effects of microbial mosquito larvicides and larvicidal oil on the development and fecundity of the tadpole shrimp *Triops newberryi* (Packard) (Notostraca:Triopsidae). J. Vector Ecol..

[12] Su T., Jiang Y., Mulla M.S. (2014). Toxicity and effects of mosquito larvicides methoprene and surface film (Agnique(R) MMF) on the development and fecundity of the tadpole shrimp *Triops newberryi* (Packard) (Notostraca:Triopsidae). J. Vector Ecol..

[13] Ferreira N.G.C., Chessa A., Abreu I.O., Teles L.O., Kille P., Carvalho A.P., Guimarães L. (2023). Toxic relationships: Prediction of TBT’s affinity to the ecdysteroid receptor of *Triops longicaudatus*. Toxics.

[14] Guimarães L., Carvalho A.P., Ribeiro P., Teixeira C., Silva N., Pereira A., Amorim J., Oliva-Teles L. (2024). Sensitivity of *Triops longicaudatus* locomotor behaviour to detect short low-level exposure to pollutants. Water.

[15] OECD (2004). Test No. 202: Daphnia *sp.* Acute Immobilisation Test.

[16] (2012). Water Quality–Determination of the Inhibition of the Mobility of Daphnia Magna Straus (Cladocera, Crustacea)-Acute Toxicity Test.

[17] Barceloux D.G. (1999). Chromium. J. Toxicol. Clin. Toxicol..

[18] Velma V., Vutukuru S.S., Tchounwou P.B. (2009). Ecotoxicology of hexavalent chromium in freshwater fish: A critical review. Rev. Environ. Health.

[19] Deng Y., Wang M., Tian T., Lin S., Xu P., Zhou L., Dai C., Hao Q., Wu Y., Zhai Z. (2019). The effect of hexavalent chromium on the incidence and mortality of human cancers: A meta-analysis based on published epidemiological cohort studies. Front. Oncol..

[20] Rao K.R., Doughtie D.G. (1984). Histopathological changes in grass shrimp exposed to chromium, pentachlorophenol and dithiocarbamates. Mar. Environ. Res..

[21] Yang G., Chen C., Yu Y., Zhao H., Wang W., Wang Y., Cai L., He Y., Wang X. (2018). Combined effects of four pesticides and heavy metal chromium (VI) on the earthworm using avoidance behavior as an endpoint. Ecotoxicol. Environ. Saf..

[22] Mishra A.K., Mohanty B. (2008). Acute toxicity impacts of hexavalent chromium on behavior and histopathology of gill, kidney and liver of the freshwater fish, *Channa punctatus* (Bloch). Environ. Toxicol. Pharmacol..

[23] Yan T., Xu Y., Zhu Y., Jiang P., Zhang Z., Li L., Wu Q. (2023). Chromium exposure altered metabolome and microbiome-associated with neurotoxicity in zebrafish. J. Appl. Toxicol..

[24] Wong C.K., Chu K.H., Tang K.W., Tam T.W., Wong L.J. (1993). Effects of chromium, copper and nickel on survival and feeding behaviour of *Metapenaeus ensis* larvae and postlarvae (Decapoda: Penaeidae). Mar. Environ. Res..

[25] Teles M., Pacheco M., Santos M.A. (2005). Physiological and genetic responses of European eel (*Anguilla anguilla* L.) to short-term chromium or copper exposure–Influence of preexposure to a PAH-like compound. Environ. Toxicol..

[26] Tallarico L.d.F., Borrely S.I., Hamada N., Grazeffe V.S., Ohlweiler F.P., Okazaki K., Granatelli A.T., Pereira I.W., Pereira C.A., Nakano E. (2014). Developmental toxicity, acute toxicity and mutagenicity testing in freshwater snails *Biomphalaria glabrata* (Mollusca: Gastropoda) exposed to chromium and water samples. Ecotoxicol. Environ. Saf..

[27] Ahmed M.K., Kundu G.K., Al-Mamun M.H., Sarkar S.K., Akter M.S., Khan M.S. (2013). Chromium (VI) induced acute toxicity and genotoxicity in freshwater stinging catfish, *Heteropneustes fossilis*. Ecotoxicol. Environ. Saf..

[28] Heffern K., Tierney K., Gallagher E.P. (2018). Comparative effects of cadmium, zinc, arsenic and chromium on olfactory-mediated neurobehavior and gene expression in larval zebrafish (*Danio rerio*). Aquat. Toxicol..

[29] Xu Y., Wang L., Zhu J., Jiang P., Zhang Z., Li L., Wu Q. (2021). Chromium induced neurotoxicity by altering metabolism in zebrafish larvae. Ecotoxicol. Environ. Saf..

[30] Wise J.P., Young J.L., Cai J., Cai L. (2022). Current understanding of hexavalent chromium [Cr(VI)] neurotoxicity and new perspectives. Environ. Int..

[31] Hellou J. (2011). Behavioural ecotoxicology, an “early warning” signal to assess environmental quality. Environ. Sci. Pollut. Res. Int..

[32] Oliva Teles L., Fernandes M., Amorim J., Vasconcelos V. (2015). Video-tracking of zebrafish (*Danio rerio*) as a biological early warning system using two distinct artificial neural networks: Probabilistic neural network (PNN) and self-organizing map (SOM). Aquat. Toxicol..

[33] Rodrigues A.P., Oliva-Teles T., Mesquita S.R., Delerue-Matos C., Guimaraes L. (2014). Integrated biomarker responses of an estuarine invertebrate to high abiotic stress and decreased metal contamination. Mar. Environ. Res..

[34] Abreu I.O., Monteiro C., Rocha A.C.S., Reis-Henriques M.A., Teixeira C., Basto M.C.P., Ferreira M., Almeida C.M.R., Oliva-Teles L., Guimaraes L. (2018). Multibiomarker interactions to diagnose and follow-up chronic exposure of a marine crustacean to Hazardous and Noxious Substances (HNS). Environ. Pollut..

[35] Colovic M.B., Krstic D.Z., Lazarevic-Pasti T.D., Bondzic A.M., Vasic V.M. (2013). Acetylcholinesterase inhibitors: Pharmacology and toxicology. Curr. Neuropharmacol..

[36] Amorim J., Fernandes M., Abreu I., Tavares F., Oliva-Teles L. (2018). *Escherichia coli*’s water load affects zebrafish (*Danio rerio*) behavior. Sci. Total Environ..

[37] Román A.C., Vicente-Page J., Pérez-Escudero A., Carvajal-González J.M., Fernández-Salguero P.M., de Polavieja G.G. (2018). Histone H4 acetylation regulates behavioral inter-individual variability in zebrafish. Genome Biol..

[38] Ellman G.L., Courtney K.D., Andres V., Feather-Stone R.M. (1961). A new and rapid colorimetric determination of acetylcholinesterase activity. Biochem. Pharmacol..

[39] Rodrigues A.P., Lehtonen K.K., Guilhermino L., Guimaraes L. (2013). Exposure of *Carcinus maenas* to waterborne fluoranthene: Accumulation and multibiomarker responses. Sci. Total Environ..

[40] Bradford M.M. (1976). A rapid and sensitive method for the quantitation of microgram quantities of protein utilizing the principle of protein-dye binding. Anal. Biochem..

[41] Ball J.W., Izbicki J.A. (2004). Occurrence of hexavalent chromium in ground water in the western Mojave Desert, California. Appl. Geochem..

[42] Bourotte C., Bertolo R., Almodovar M., Hirata R. (2009). Natural occurrence of hexavalent chromium in a sedimentary aquifer in Urânia, State of São Paulo, Brazil. An. Acad. Bras. Ciênc.

[43] Tziritis E., Kelepertzis E., Korres G., Perivolaris D., Repani S. (2012). Hexavalent chromium contamination in groundwaters of Thiva Basin, central Greece. Bull. Environ. Contam. Toxicol..

[44] WHO (2003). Guidelines for Drinking-Water Quality.

[45] Moreira R.A., da Silva Mansano A., Rocha O. (2015). The toxicity of carbofuran to the freshwater rotifer, *Philodina roseola*. Ecotoxicology.

[46] Wu Y., Lin C., Yuan L. (2007). Characteristics of six cladocerans in relation to ecotoxicity testing. Ecol. Indic..

[47] Castro B.B., Freches A.R., Rodrigues M., Nunes B., Antunes S.C. (2018). Transgenerational effects of toxicants: An extension of the *Daphnia* 21-day Chronic Assay?. Arch. Environ. Contam. Toxicol..

[48] Peluso L., Giusto A., Rossini G.D.B., Ferrari L., Salibian A., Ronco A.E. (2011). *Hyalella curvispina* (AMPHIPODA) as a test organism in laboratory toxicity testing of environmental samples. Fresenius Environ. Bull..

[49] Obregón-Barboza H., Maeda-Martínez A.M., Murugan G. (2001). Reproduction, molting, and growth of two Mexican uniparental forms of the tadpole shrimp *Triops* (Branchiopoda:Notostraca) under a recirculating culture system. Hydrobiologia.

[50] Arzate-Cardenas M.A., Martinez-Jeronimo F. (2012). Energy resource reallocation in *Daphnia schodleri* (Anomopoda: Daphniidae) reproduction induced by exposure to hexavalent chromium. Chemosphere.

[51] Guimarães L., Guilhermino L., Afonso M.J., Marques J.M., Chaminé H.I. (2019). Assessment of urban groundwater: Towards integrated hydrogeological and effects-based monitoring. Sustain. Water Resour. Manag..

[52] Ribeiro S., Guilhermino L., Sousa J.P., Soares A.M. (1999). Novel bioassay based on acetylcholinesterase and lactate dehydrogenase activities to evaluate the toxicity of chemicals to soil isopods. Ecotoxicol. Environ. Saf..

[53] Diamantino T.C., Guilhermino L., Almeida E., Soares A.M. (2000). Toxicity of sodium molybdate and sodium dichromate to *Daphnia magna* straus evaluated in acute, chronic, and acetylcholinesterase inhibition tests. Ecotoxicol. Environ. Saf..

[54] Gutierrez M.F., Gagneten A.M., Paggi J.C. (2012). Exposure to sublethal chromium and endosulfan alter the diel vertical migration (DVM) in freshwater zooplankton crustaceans. Ecotoxicology.

[55] Gutierrez M.F., Paggi J.C., Gagneten A.M. (2012). Infodisruptions in predator-prey interactions: Xenobiotics alter microcrustaceans responses to fish infochemicals. Ecotoxicol. Environ. Saf..

